# Beta 2 glycoprotein I and neutrophil extracellular traps: Potential bridge between innate and adaptive immunity in anti-phospholipid syndrome

**DOI:** 10.3389/fimmu.2022.1076167

**Published:** 2023-01-09

**Authors:** Claudia Grossi, Nagaja Capitani, Marisa Benagiano, Cosima Tatiana Baldari, Chiara Della Bella, Paolo Macor, Francesco Tedesco, Maria Orietta Borghi, Norma Maugeri, Mario Milco D’Elios, Pier Luigi Meroni

**Affiliations:** ^1^ Istituto Auxologico Italiano, Istituto di Ricovero e Cura a Carattere Scientifico (IRCCS), Laboratory of Immuno-Rheumatology, Milan, Italy; ^2^ Department of Experimental and Clinical Medicine, University of Florence, Florence, Italy; ^3^ Department of Life Sciences, University of Siena, Siena, Italy; ^4^ Department of Life Science, University of Trieste, Trieste, Italy; ^5^ Department of Clinical Science and Community Health, University of Milan, Milan, Italy; ^6^ Autoimmunity and Vascular Inflammation Unit, Division of Immunology, Transplantation & Infectious Diseases, Istituto di Ricovero e Cura a Carattere Scientifico (IRCCS) San Raffaele Institute, Milan, Italy; ^7^ Department of Molecular and Developmental Medicine, University of Siena, Siena, Italy

**Keywords:** β2GPI, neutrophils, T cells, NETs, APS, aPL, inflammation, thrombosis

## Abstract

Antiphospholipid syndrome (APS) is a systemic autoimmune disorder characterized by recurrent vascular thrombosis and miscarriages in the absence of known causes. Antibodies against phospholipid-binding proteins (aPL) are pathogenic players in both clotting and pregnancy APS manifestations. There is sound evidence that antibodies specific for beta2 glycoprotein I (β2GPI) trigger thrombotic and pregnancy complications by interacting with the molecule on the membranes of different cell types of the coagulation cascade, and in placenta tissues. In addition to the humoral response against β2GPI, both peripheral and tissue CD4^+^ β2GPI-specific T cells have been reported in primary APS as well as in systemic lupus erythematosus (SLE)-associated APS. While adaptive immunity plays a clear role in APS, it is still debated whether innate immunity is involved as well. Acute systemic inflammation does not seem to be present in the syndrome, however, there is sound evidence that complement activation is crucial in animal models and can be found also in patients. Furthermore, neutrophil extracellular traps (NETs) have been documented in arterial and venous thrombi with different etiology, including clots in APS models. Keeping in mind that β2GPI is a pleiotropic glycoprotein, acting as scavenger molecule for infectious agents and apoptotic/damaged body constituents and that self-molecules externalized through NETs formation may become immunogenic autoantigens, we demonstrated β2GPI on NETs, and its ability to stimulate CD4^+^β2GPI-specific T cells. The aim of this review is to elucidate the role of β2GPI in the cross-talk between the innate and adaptive immunity in APS.

## 1 Introduction

Anti-Phospholipid Syndrome (APS) is an autoantibody-mediated vasculopathy characterized by recurrent arterial/venous thrombosis and/or miscarriages (1). There is evidence that antibodies against phospholipid-binding proteins (aPL) are pathogenic in addition of being diagnostic/classification markers (2). In particular, antibodies specific for beta2 glycoprotein I (β2GPI) may trigger clotting and pregnancy complications by interacting with the molecule on the membranes of cells of the coagulation cascade and of placenta tissues. In addition to the humoral response against β2GPI, both peripheral and tissue CD4^+^ β2GPI-specific T cells have been reported in primary APS as well as in systemic lupus erythematosus (SLE)-associated APS (3). Besides their helper role for B cell activation, these cells have been involved in the formation of the atherosclerotic plaques associated with APS and in modulating their instability (4, 5).

While adaptive immunity plays a clear role in APS, innate immunity is emerging as another player. For example, complement activation is crucial in animal models and can be demonstrated also in patients (6). Despite this finding, acute systemic inflammation does not seem to be the main characteristic of the syndrome (7), and the cross-talk between complement and the coagulation cascade was suggested to be more important in APS thrombotic vasculopathy (8, 9). The lack of a clear inflammatory signature in placentas from obstetric APS is consistent with a similar non inflammatory pathogenesis (10). The description of the involvement of cells of the innate immunity, such as monocytes, and their increased tissue factor (TF) membrane expression in APS is consistent with the thrombophilic state in these patients (11, 12). Moreover, β2GPI is naturally present on neutrophils, more than on monocytes, both in APS patients and healthy controls, and neutrophil extracellular traps (NETs) have been recently shown to contribute to the coagulation cascade and to be an integral component of arterial and venous thrombi with different etiology, including APS clots (13–15).

In this review, we will discuss the state of the art and we will present new data on the role of neutrophils as innate immunity effector cells in APS.

## 2 The key role of membrane β2GPI in APS pathogenesis

Β2GPI is an abundant plasma protein highly conserved across the animal kingdom, suggesting important biological functions (16, 17). Despite the involvement of the molecule in several biological pathways (**Table 1**), the specific biological role of β2GPI is still a matter of research (17, 18, 25). Nevertheless, β2GPI is widely accepted as the main antigenic target for aPL both in APS laboratory diagnostic assays and pathogenic mechanisms (2, 17).

**Table 1:** β2GPI pleiotropic role.

Biological Context	Role	References
Coagulation	Anti- and Pro-Coagulant Activity	(17–19)
Complement	C3/C3b and MBL Interaction	(8, 20, 21)
Apoptosis	Scavenger Molecule Binding to Apoptotic Material	(20, 22)
Oxidation-Reduction Reactions	Regulation of Oxidative Stress-Induced Cell Injury	(23)
Ischemia-Reperfusion Injury	Vascular Injury and Phagocytosis of Apoptotic Neurons in Experimental Brain Stroke	(20)
Dendritic Cells	DC Maturation and Function Modulation	(24)
Innate Immunity	First Defence Intervention and Switch from Innate to Adaptive Immunity	(18, 25)

B2GPI binds to several receptors expressed on different cell types involved in the coagulation cascade and placentation, and can be recognized by circulating aPL. Once bound, the antibodies may crosslink the β2GPI-receptor complex and trigger intracellular signaling leading to cell responses crucial for the clinical manifestations (2). For example, the typical vascular APS is characterized by thrombotic events in well-localized medium/large vessels. There is sound evidence that β2GPI-dependent aPL induce an endothelial perturbation with the expression of a pro-inflammatory and pro-coagulant phenotype suggesting that endothelial perturbation/activation is likely the initiating step. Once activated, the endothelium may involve other circulating cells downstream such as monocytes, neutrophils, and platelets, all contributing to clotting. Complement activation is a hallmark of APS with increased circulating levels of activation products and complement tissue deposition (6). Despite this, an inflammatory signature is not characteristic of vascular APS supporting the idea that the crosstalk of complement with the coagulation cascade is playing a more critical role (26).

On the other hand, the catastrophic APS (CAPS), being systemic thrombotic microangiopathy, displays different pathogenesis and affects small vessels in several anatomical sites with massive production of pro-inflammatory cytokines and the involvement of platelets and neutrophils (27).

Β2GPI-dependent aPL are also associated with obstetric APS manifestations and their pathogenic role is supported by several *in vitro* and *in vivo* models. B2GPI can be found on placental and decidual tissues, it binds to its cell membrane receptors and in addition, its cationic PL-binding site in the fifth domain interacts with the anionic charges of the syncytiotrophoblast cell membranes (1). The β2GPI-receptor complex is recognized by maternal β2GPI-dependent aPL which mediate defective placentation eventually responsible for miscarriages (1).

## 3 Neutrophils, NETs and APS

As a result of intense work over the past decade, the molecular basis of immunothrombosis in APS is starting to be elucidated (28). Neutrophils are an essential component of innate immunity. Phagocytosis, the production of reactive oxygen species, the release of proteases and the generation of extracellular traps (NETs) are effector mechanisms that neutrophils rely on to prevent infections, eliminate pathogens and control bystander inflammatory vascular damage (29, 30). NETs however have been documented in arterial and venous thrombi with different etiology (31–33).

NETs consist of lattices of extracellular DNA, decorated with citrullinated histones and neutrophil proteases (29, 34, 35). Various components might be involved in the pro-coagulant effects of NETs. Cell-free DNA activates the coagulation cascade through the intrinsic pathway acting on factors XII and XI (36, 37). Extracellular histones promote thrombin generation by activating platelets (38) and by impairing thrombomodulin-dependent protein C activation (39). The extrinsic pathway of coagulation seems to be activated by the presence of bioactive tissue factor along NETs (40).

The pro-thrombotic action of NETs could be relevant for the pathogenesis of APS. An increase in the plasma concentration of NET by-products has in fact been documented in patients with vascular APS (41). The increase may reflect a higher NET generation, a lower NET degradation rate or both (42).

The presence of neutrophils directly involved in the generation of NETs in APS patients has been suggested by the detection of “Low-Density Granulocyte” (LDG), a population originally described in SLE patients as responsible for spontaneous NET release (43, 44). Furthermore, classical “high-density” neutrophils from APS patients are more efficient than those from healthy subjects in generating NETs (45, 46), probably indicating that a previous *in vivo* priming occurred.

The three-dimensional structure offered by NETs absorbs and traps exogenous molecules, including the humoral innate immune response players. This has been well documented for complement (28). There is evidence that β2GPI is also expressed by circulating phagocytes regardless of their state of activation (13). As such it could represent a target antigen recognized by anti-β2GPI antibodies. The interaction of the aPL with the β2GPI at the neutrophil membrane could determine an activation signal, stimulating and amplifying the generation of NETs (13, 15, 47)

One model of fetal loss induced by the passive infusion of very high amounts of aPL raised the potential role of neutrophil recruitment and activation by C5a and/or Tumor Necrosis Factor (TNF) in aPL-mediated placenta damage (48). This finding may suggest increased NETosis which in turn can contribute to damage of trophoblast, decidual and endothelial cells. A recent study by Lu et al. (14) reported elevated circulating levels of cell-free DNA and NETs in sera of pregnant women with APS indirectly supporting the possible pathogenic role of NETosis. However, a direct histological demonstration of increased NETosis in APS placentas is still lacking, even though Marder et al. (49) reported that the number of neutrophils, neutrophil NETs and the ratio of NETs/total neutrophils are increased in placentas from SLE patients with miscarriages, in particular, if aPL are present.

The potential interaction NETs-β2GPI in vascular and obstetric APS is outlined in **Supplementary Table 1** and in **Supplementary Figure 1**.

## 4 B2GPI co-localizes with NETs and is recognized by β2GPI-T cell clones

**Figure 1:**
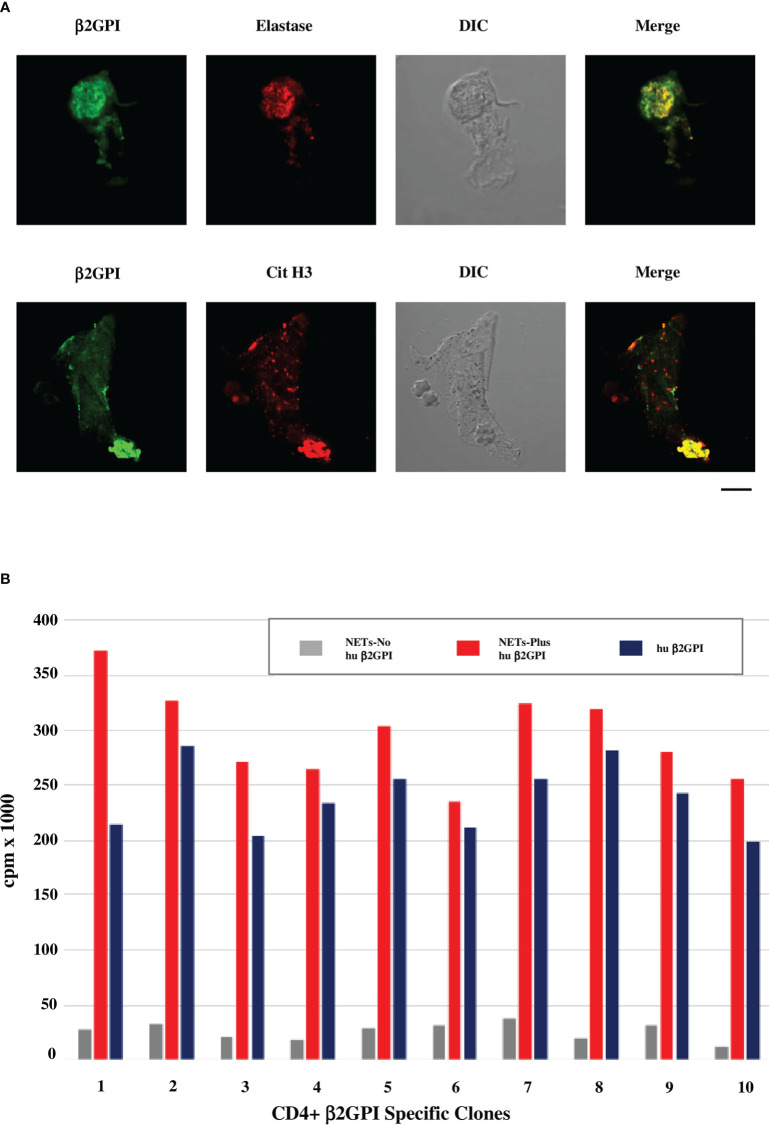
β2GPI co-localizes with NET markers and β2GPI-containing NETs stimulate proliferation of SLE-APS derived T cell clones. **(A)** Confocal microscopic analysis of human neutrophils cultured in media with human AB serum and stimulated with PMA for production of NETs. Green staining identifies β2GPI, detected by MBB2 antibody (50), red staining identifies elastase by rabbit polyclonal anti-neutrophil elastase antibodies (Abcam) in the upper panel or citrullinated histone H3 (Cit H3) by IgG monoclonal anti-citrullinated histone H3 antibodies (LS-Bio) in the lower panel. Differential Interference Contrast (DIC) was applied as a non-destructive contrast enhancing technique to locate a specific area of interest in the specimen. Briefly, human neutrophils were purified from peripheral blood of healthy donors (EasySep Human Neutrophil isolation kit, Stemcell Technologies) and cultured in medium RPMI 1640 with 5% human serum (Sigma Aldrich) as source of β2GPI, or in β2GPI-free medium (RPMI 1640 in the absence of any source of β2GPI). The cells were stimulated by 100nM PMA for 2h at 37°C for the production of NETs and analyzed for the presence of β2GPI by IF, using the human MBB2 MoAb IgG against D1-β2GPI (50). Co-localization of β2GPI with NET markers, such as elastase and histone H3, was investigated by confocal microscope (Zeiss LSM700 from Carl Zeiss). Alexa Fluor 488- and 555-labeled secondary antibodies were used: Alexa Fluor™ 488-Goat anti-Mouse IgG (H+L) Cross-Adsorbed Secondary Antibody and Alexa Fluor™ 555-Goat anti-Rabbit IgG (H+L) Cross-Adsorbed Secondary Antibody (Life Technologies). Scale bar 10 mm. **(B)** Proliferative response of SLE-APS patient derived β2GPI-specific T-cell clones to NETs, containing or not human β2GPI, or to human purified β2GPI alone (10 nM). Human β2GPI was purified as previously described (51). T-cell clones were derived from the atherosclerotic plaques of the patients, as previously described (4, 5). Further methodological details are reported in the **Supplementary Material**.

**Figure 1A** shows that β2GPI binds to NETs obtained from neutrophils purified from healthy donors and cultured in presence of human serum as a source of β2GPI. The binding of β2GPI to NETs was assessed by indirect immunofluorescence (IIF) and confocal microscopy using MBB2 monoclonal IgG specific for the D1 of the molecule (50) and antibodies against elastase or citrullinated histone H3 as NET markers. Moreover, β2GPI containing NETs significantly stimulate the proliferation of SLE-APS derived β2GPI-specific CD4^+^ T cell clones, more efficiently than native human purified β2GPI (**Figure 1B**). The reactivity of MBB2 monoclonal IgG supports the exposure of D1 immunodominant epitope of β2GPI, as reported when the molecule binds to anionic structures (17). We previously reported that β2GPI-specific CD4^+^ T cell clones from PAPS atherosclerotic plaques are more reactive to D1 than to D4,5 (4). The data presented are consistent with this finding and support that the β2GPI co-localizing with NETs offers immunodominant epitopes.

The study was approved by the Ethics Committee at Istituto Auxologico Italiano (22_07_2010) and the patients gave their informed consent.

## 5 NET degradation

Under physiological conditions NETs degrade rapidly, an event crucial to quench their potential to damage bystander tissues. Endogenous DNases play an important role in the dismantling of NETs (29, 42, 52). However, DNases only cleave NETs, allowing the release of proteases and histones. In the circulation these molecules, although isolated from the NETs, will retain their ability to damage the vascular wall (29, 53). Another mechanism of NET elimination is phagocytosis by macrophages. The extent of phagocytosis could be accelerated by resolvins, thus contributing to the action of this group of molecules in terminating inflammation (54).

Defective NET degradation could further support the endothelial perturbation leading to the thrombophilic phenotype in APS. Preliminary data showed that NET degradation is impaired in APS patients and this effect is related to antibodies against NETs, which do not correlate with aPL (55).

## 6 Therapeutic strategies targeting NETs

Strategies specifically designed to interfere with NETs are not yet available. It is however possible that the benefit associated with some anti-inflammatory treatments is at least partially due to the modulation of NET production or biological activity. Low molecular weight heparins (LMWH) are currently used, mostly on an empirical basis, in conditions in which NETs play a critical role. The clinical benefit, that is independent of the anticoagulant action, has been demonstrated in controlled studies in patients with asthma, chronic obstructive pulmonary disease, inflammatory bowel disease, and COVID-19 (56, 57). LMWH has also emerged as an option for the prevention of complications of pregnancy in women at high risk of recurrence (58–60) and is a cornerstone in the treatment of pregnancy complications associated to APS (58, 61).

Several mechanisms are likely to be involved in the anti-inflammatory action of LMWH, including its effect on neutrophils and neutrophil interaction with the vessel wall and platelets (62). Moreover, LMWH can dismantle NETs that have already been organized (63) while inducing a profound change in the ability of human neutrophils to generate NETs and to mobilize the content of the primary granules in response to unrelated inflammatory stimuli (64–66). Moreover, heparin was shown to displace tissue-bound β2GPI because of its stronger avidity for the molecule (67). This last pharmacological mechanism could represent an additional direct effect of heparin on the availability of β2GPI on NETs.

## 7 Discussion

The pathogenesis of APS is becoming more and more complex in the last years involving different mechanisms that are likely to play a diverse role depending on the clinical variants of the syndrome. In particular, evidence of the interplay between adaptive and innate immunity is emerging.

Vascular APS is characterized by clotting in localized areas of the arterial or the venous tree at variance with CAPS, in which systemic thrombotic microangiopathy is responsible for the clinical picture. Endothelial perturbation is apparently the initial pathogenic step and it may explain the clinical manifestations associated with classical vascular APS. However, endothelial activation involves other cells downstream, particularly platelets and neutrophils.

A lot of attention has been recently paid to the role of neutrophils in APS as an example of the interplay with innate immunity. We have previously discussed the evidence for the involvement of neutrophil activation and NET formation in APS from both animal models and from studies in patients (13–15).

We describe in this paper the ability of β2GPI to interact with NETs and demonstrate that the bound molecule displays the epitope characteristics showed by β2GPI complexed with anionic structures (17). Moreover, β2GPI-NETs complexes stimulate β2GPI-specific CD4^+^ T cells more efficiently than the circulating naïve molecule. This mechanism may be responsible for offering self β2GPI to autoreactive β2GPI specific T helper cells eventually leading to loss of tolerance, β2GPI specific B cell clonal expansion and aPL production in genetically predisposed subjects. Infections and/or inflammatory events are relatively frequent, may induce neutrophil activation and NETosis and ultimately can be responsible for β2GPI binding to NETs.

Once the anti-β2GPI response is started, aPL can react with β2GPI on neutrophils as described (13, 15, 47). This may activate neutrophils directly, boost inflammation and force neutrophils to act as antigen-presenting cells *via* MHC II-mediated pathways, further stimulating the adaptive response against β2GPI (68). In addition, neutrophils can be indirectly activated *via* complement activation leading to further NET formation and β2GPI binding. The mutual interactions among the complement and coagulation systems may eventually contribute to thrombotic events in vascular APS (69).

Mitochondrial NETosis offers another potential linkage between β2GPI and NETs. Mitochondria exert a peculiar role in neutrophils, being ejected outside the cell engaging bacteria, an early event in the antimicrobial response (70). The mitochondrial membrane contains cardiolipin (CL) as a unique phospholipid that plays a central role in mitochondrial function and dynamics (71). We may speculate that mitochondria in the extracellular milieu could lead to unusual CL exposure and allow the interaction between CL and β2GPI, naturally present on neutrophils (13).

The presence of circulating aPL may affect all these systems. For example, NETs could not be regularly cleared by β2GPI-dependent scavenging mechanisms because of aPL interference. The reported impaired NET degradation in APS patients associated with the presence of antibodies against NETs is consistent with this effect (55).

It is likely that apoptotic blebs and NETs may interplay at the sites of inflammation.

Their clearance systems partially overlap (72). β2GPI plays a role in scavenging LPS and apoptotic blebs, and at the same time is able to bind nucleic acids *via* the cationic binding site in the fifth domain, these being good starting points for a candidate protein involved in NET clearance (20, 22, 30, 72). Once again, β2GPI-dependent aPL may interfere with these mechanisms. An additional pro-thrombotic effect of NETs has been recently associated with activated protein C resistance in APS (73).

In conclusion, there is emerging evidence that β2GPI is at crossroads between adaptive and innate immunity in APS pathogenesis and may represent a potential target for new therapies. Consistently, synthetic peptides competing with the PL-binding site and able to inhibit the binding of β2GPI to physiological targets showed a protective effect in animal models (74–76).

## Data availability statement

The raw data supporting the conclusions of this article will be made available by the authors, without undue reservation.

## Ethics statement

The study was approved by the Ethics Committee at Istituto Auxologico Italiano (22_07_2010). The patients/participants provided their written informed consent to participate in this study.

## Author contributions

MMD'E, PLM contributed to conception and design of the study, NC, MB, CTB, CDB performed the experiments and organized the database, CG, PM, FT, prepared purified β2GPI and MBB2. CG, MOB, NM, PLM wrote the first draft of the manuscript. All authors contributed to manuscript revision, read, and approved the submitted version.
