# Supplementary material for: Beta 2 glycoprotein I and neutrophil extracellular traps: Potential bridge between innate and adaptive immunity in anti-phospholipid syndrome
Source: Front Immunol. 2023 Jan 9;13:1076167. doi: 10.3389/fimmu.2022.1076167 (PMC9868732; doi:10.3389/fimmu.2022.1076167)
Supplement: Supplementary file 1 [file DataSheet_1.pdf]

Supplementary Material

Supplementary Table 1. Evidences of NETs in APS

NETosis in APS patients	Reference	Potential role of β_2GPI?
Increase of circulating cell-free DNA and NETs	Yalavarthi S et al, doi: 10.1002/art.39247	Scavenging
Presence of β_2GPI on neutrophils	Yalavarthi S et al, doi: 10.1002/art.39247	Modulation of NETotic neutrophils clearance
APS neutrophils prone to release of NETs	Yalavarthi S et al, doi: 10.1002/art.39247<br>Lu Y et al, doi: 10.1111/jcmm.15321	Neutrophils activation by anti-β_2GPI antibodies
Degradation of NETs impaired by anti-NET antibodies	Zuo Y et al, doi: 10.1002/art.41460	Contribution to NETs clearance impairment mediated by anti-β_2GPI antibodies
Increased cell-free DNA and MPO-DNA complexes circulating levels in pregnant APS	Lu Y et al, doi: 10.1111/jcmm.15321	Scavenging
Correlation of circulating MPO-DNA complexes and cell-free DNA with APC resistance	Foret T et al, doi:10.1093/rheumatology/<br>keab853	APC inhibition

**NETosis in *in vitro* and *in vivo* APS models**	**Reference**	**Potential role of β_2GPI?**
Anti-β_2GPI antibodies induce release of NETs by normal neutrophils	Yalavarthi S et al, doi: 10.1002/art.39247<br><br>Lu Y et al, doi: 10.1111/jcmm.15321	Neutrophils priming by anti-β_2GPI antibodies
aPL prime circulating neutrophils	Meng H et al, doi: 10.1002/art.39938	Neutrophils priming by anti-β2GPI antibodies

NETs: neutrophil extracellular traps; APS: antiphospholipid syndrome; β_2GPI: beta 2 glycoprotein I; MPO: myeloperoxidase; APC: activated protein C; aPL: antiphospholipid antibodies

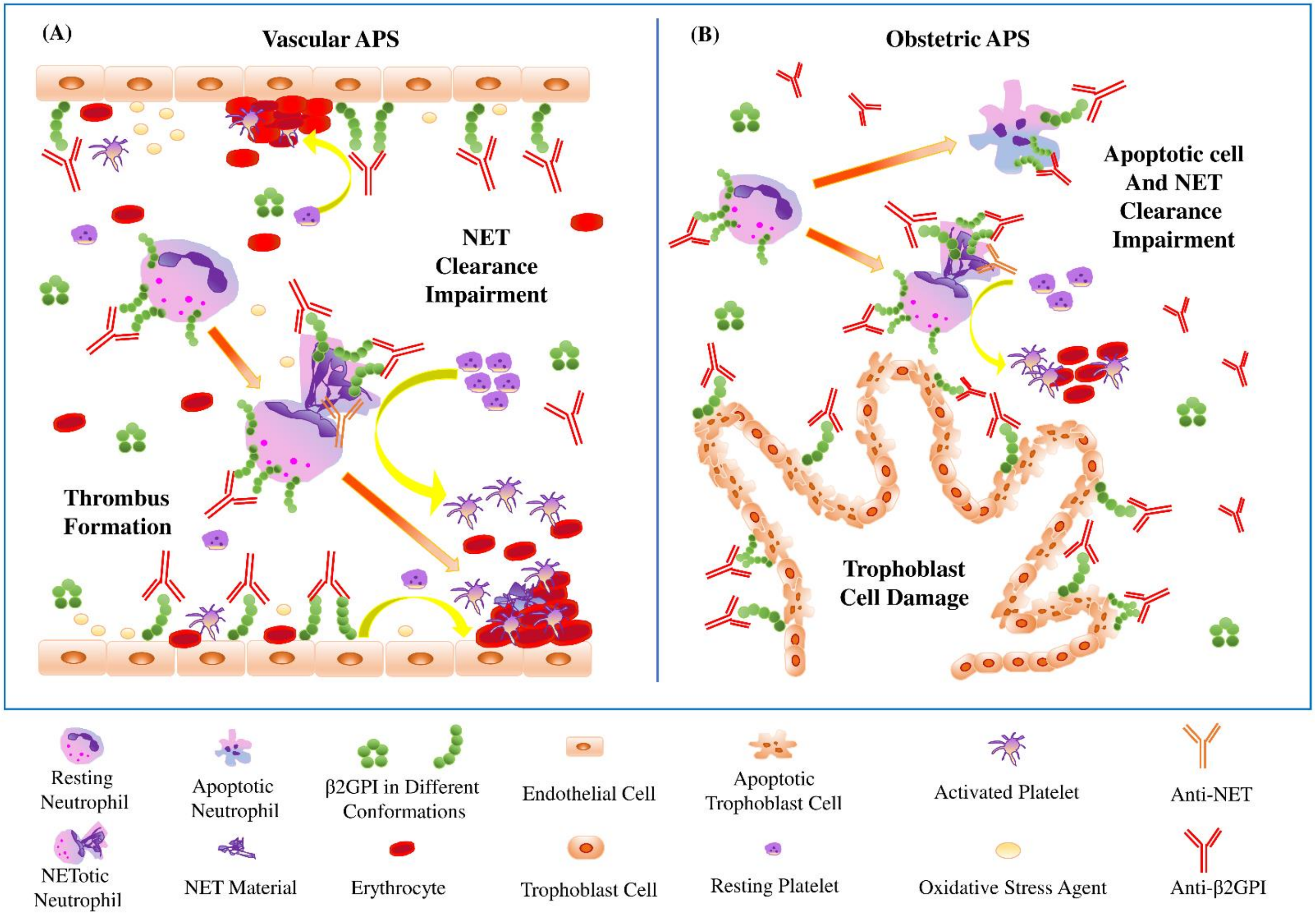

Supplementary Figure 1. Possible interactions of β2GPI and neutrophil NETs in vascular (A) and obstetric (B) APS. (A) Anti-β2GPI antibodies may react with β2GPI upregulated on the endothelium perturbed by the 2nd hit (e.g. an oxidative stress agent), further enhancing endothelial activation. Neutrophils can then be recruited/activated as a downstream effect, or can be directly activated by antibodies against surface β2GPI, supporting an increased NETosis which eventually contributes to clotting. **(B)** Neutrophils recruitment/activation are triggered by production of TNF and C5a in the placenta. Activated neutrophils may display an increased NETosis which can contribute to the damage of trophoblast and decidual cells, as well as of placental endothelium. In both the situations, antibodies bound to β2GPI present on NETs and anti-NET antibodies may form a sort of protective shield making NETs clearance less efficient and ultimately fueling the pathogenic process.

Supplementary Methods

Proliferative response of CD4+-T cell clones to NETs

NETs from the peripheral blood of healthy donors were generated *in vitro* according to O'Meara (Meara, CHO. et al. Nat Commun.2020; 11: 6408. doi:10.1038/s41467-020-20231-y). Neutrophils were isolated using the EasySep Human Neutrophil isolation kit, (Stemcell Technologies) and plated in 12-well plates (Costar/Corning) at 1.5×10^6 cells/well in 500 µL/well medium MCDB 131/0.5% BSA. PMA 50 nM (Sigma) was added to plated neutrophils before incubation at 37 °C, 5% CO_2 for 4 hrs to induce β2GPI-free NET formation. In order to have β2GPI-NETs, 5% human serum (Sigma Aldrich) was used as a source of β2GPI. The presence or absence of β2GPI was evaluated by indirect immunofluorescence using the human IgG monoclonal antibody MBB2 against D1-β2GPI. After incubation, supernatants were carefully collected and the remaining neutrophil/NETs were gently washed twice with 1 mL PBS to remove remaining PMA. To detach NETs from cell debris, NETs were digested in the well with 4 U/mL ALU1 (New England BioLabs Inc.) in 400 µL/well MCDB 131/0.5% BSA at 37 °C for 20 min. Digested NETs were collected by vigorously mixing and subsequent centrifugation at 300x*g* for 5 min at 4 °C to separate out remaining cell debris. Cell-free supernatant containing soluble NETs or β2GPI-NETs were collected and stored at -20 °C until use. $CD4^+$ T-cell clones were obtained from the atherosclerotic plaques of SLE-APS patients, as previously described (Benagiano et al, Haematologica. 2019;104(12):2519-2527. doi: 10.3324/haematol.2018.209536). $CD4^+$ T-cell blasts (10^5/well) were stimulated for 60 h in the presence of purified human β2GPI (10 nM), or NETs containing or not β2GPI (vol/vol), and irradiated autologous monocytes (0.5×10^5). At 16 hrs before harvesting, 0.5 µCi of (^{3}H)dT (Amersham Pharmacia Biotech) were added, and radionuclide uptake was measured in a β-counter. The mitogenic index (MI) was calculated as the ratio between mean values of cpm obtained in stimulated cultures and those obtained in the presence of medium alone.
